# Supplementary material for: Are carbon emissions trading and green financial instruments synergistic? -Comprehensive quantitative research based on content analysis
Source: PLoS One. 2024 Mar 7;19(3):e0298601. doi: 10.1371/journal.pone.0298601 (PMC10919601; doi:10.1371/journal.pone.0298601)
Supplement: S1 Data — (ZIP) [file pone.0298601.s001.zip › Data/地级市Z_F工作报告.zip!/╜¡╦╒/│ú╓▌╩╨/└┤╫╘╣1⁄2╓┌║┼í╛┬φ┐╦╩2╛▌═°í┐_2019.docx]

各位代表：

　　现在，我代表常州市人民政府向大会报告政府工作，请予审议，并请市政协各位委员和其他列席人员提出意见。

　　一、2018年工作回顾

　　过去一年，面对严峻复杂的外部环境和艰巨繁重的改革发展任务，市政府在中共常州市委的坚强领导和市人大、市政协的监督支持下，团结依靠全市人民，深入学习贯彻习近平新时代中国特色社会主义思想和党的十九大精神，坚持新发展理念，紧扣高质量发展要求，扎实推进种好幸福树、建好明星城各项工作，较好地完成了市委十二届五次、六次全会和市十六届人大二次会议确定的各项任务，高质量发展实现良好起步。

　　一年来，全市经济社会运行总体平稳、稳中有进、稳中向好。一是主要指标平稳增长。完成地区生产总值7050.27亿元，增长7%；固定资产投资增长7.5%，其中工业投资增长7.1%；完成进出口总额2266.4亿元，增长7%；实际到账外资26.11亿美元；社会消费品零售总额增长9.1%。二是质量效益明显提升。完成税收收入915.17亿元、增长10.4%，总量前进一位、列全省第四；一般公共预算收入560.3亿元、增长8%，税比87.3%、全省第二；规上工业企业利润总额增长19.1%，其中民营工业企业利润总额增长20%；高新技术产业产值占比达47%以上；高新技术产业投资增长13.3%。三是创新活力显著增强。全社会研发经费占比达2.81%，科技进步贡献率达63.8%，万人发明专利拥有量超32.5件，实现中国专利金奖零的突破，新增高新技术企业280家、市级以上企业研发机构220家，科教城连续5年荣膺中国最佳创业园区第二名。四是人民生活不断改善。全体居民人均可支配收入增长8.5%，城镇登记失业率控制在3%以内，物价保持平稳运行，居民消费价格涨幅控制在2.2%左右。30件民生实事扎实推进，解决了安阳里危积陋改造等一批百姓关注的操心事烦心事。全面小康指数位居江苏第四、全国第十二位。

　　一年来，我们迎难而上、主动作为，凝心聚力、攻坚克难，专注干好自己的事，主要做了以下工作：

　　（一）聚焦提质增效，实体经济呈现新发展。抓牢实体经济这个根本，突出质量效益导向，全力以赴抓转型、调结构。扎实开展“重大项目增效年”活动，新增签约30亿元或3亿美元以上重大项目20个，其中时代新能源三期等3个项目超100亿元或10亿美元，新恒东光学薄膜一期、蒂森克虏伯汽车转向柱等41个项目竣工。深入实施“三位一体”工业转型升级战略，十大产业链产值增长12.8%、占比达42%，新能源汽车及零部件、新一代信息技术、智能制造装备产业产值分别增长24.2%、35.3%、19.8%。全域旅游加快推进，接待游客7244.91万人次、增长9.8%，旅游总收入1088.64亿元、增长14.2%；溧阳曹山获批省级旅游度假区。创新发展现代服务业，常州高新区成为省级服务业综合改革试点，武进绿建区等3家单位获评省生产性服务业集聚示范区，万帮充电获批国家中小企业公共服务示范平台，检验检测认证产业园创成国家检验检测高技术服务业集聚区。稳步发展现代农业，国家现代农业示范区建设水平位居全国前列，国家农产品质量安全市创建成效显著。粮农产品生产供应稳定，供销社综合改革不断深化。扎实推进质量强市建设，中国驰名商标累计107件、全省第二。

　　（二）聚焦创新驱动，转型升级实现新突破。坚持把创新作为引领发展的第一动力，持续推进苏南国家自主创新示范区建设。双创平台建设加快推进，引进公共研发创新平台3家、新型研发机构4家，新增省市共建重大科技载体平台项目5个，新能源汽车能源与信息创新中心、石墨烯创新中心获批省级制造业创新中心试点，新增省级以上众创空间、孵化器、加速器等科技创业平台23家。企业创新主体地位加快提升，天合光能和上上电缆获第五届中国工业大奖、武进不锈获提名奖，5家企业中标国家工业强基工程项目、累计15家，均居全国地级市第一；常州大学等单位参与的3个项目获国家科学技术进步二等奖；获评工信部单项冠军4个、省隐形冠军企业5家，新增11家省级工业设计中心、工业互联网四星以上上云企业13家，均居全省第一。积极培育发展“四新经济”，航天云网、中机云创、苏文电能、天正工业互联网等新业态新模式加速发展，国家健康医疗大数据中心与产业园加快建设。深入实施“龙城英才计划”，引进创业人才项目247个、各类高层次紧缺人才600多名，每万名劳动者中高技能人才数连续五年全省第一。大力推进企业上市，新增上市企业4家，总数达58家；新增新三板挂牌企业10家，总数达113家。

　　（三）聚焦改革开放，发展环境得到新优化。坚持把改革开放作为“关键一招”，全面深化重点领域改革，提升双向开放水平，位居福布斯2018年中国大陆最佳地级城市30强第四位。产城融合综合改革稳步推进，探索形成综合提升型、专业集聚型、公共服务优化型等融合发展路径。落实中央和省统一要求，稳步推进政府机构和事业单位改革。继续深化“放管服”改革，大力推进“一网通办”，在全省率先实现工商登记和不动产登记“一城通办”，高质量实现“3550”改革目标。大力支持民营经济发展，表彰改革开放40年50位杰出民营企业家，出台系列政策措施，兑现各类减免税优惠超500亿元；强化金融服务功能，新增贷款841亿元、历史最多，设立50亿元产业投资引导基金；积极防范金融风险，主动化解重点企业资金链问题，成立首期30亿元的上市公司纾困基金。深入推进市属国资国企改革发展，在全省率先出台三年行动计划，明确差异化发展定位，完成新一轮战略性重组。有序推进农村承包地“三权分置”、集体产权制度改革，溧阳市实施土地综合整治新增耕地2.47万亩；武进区顺利完成国家农村土地制度“三项改革”试点任务，集体经营性建设用地入市8万亩，走在全国前列。全面推进工业企业资源集约利用综合评价，建设用地节约集约利用综合指数位居全国前十。积极应对中美贸易摩擦，推出“九法联用”举措，外资外贸保持平稳增长，制造业实际到账外资增长58.2%。积极推进跨境贸易便利化，海关特殊监管区域货物通关时间压缩三分之一。常州国际机场年旅客吞吐量突破330万人次。大力推进园区平台高质量发展，常州经开区通过三年奋进成为全市经济重要增长极，江苏中关村获批省级高新区，苏澳合作园区建设稳步推进，新增省级特色小镇3家。

　　（四）聚焦绿色发展，生态建设取得新进展。坚持生态优先、绿色发展，严格落实“263”专项行动，全力做好中央环保督察“回头看”和省级环保督察交办问题整改，切实解决了一大批生态环境问题。认真落实长江经济带“共抓大保护、不搞大开发”要求，编制完成沿江地区绿色转型发展规划，出台推动长江经济带高质量发展走在前列实施方案，统筹推进长江生态安全带、太湖生态保护圈、武进生态保护引领区建设。组织开展大气污染防治攻坚行动，全年完成重点治气工程1835项，专项整治化工、“散乱污”企业（作坊）、成品油取得阶段性成果，关停化工企业205家，圆满完成进博会等重大活动空气质量保障任务。坚持科学治污、精准治污，开展重点区域污染源走航监测、绿色工业云精准监测，实施企业限停产豁免制度。全面落实河长制、湖长制，主城区畅流活水工程完成年度目标任务，省考以上断面水质优Ⅲ类比例达60.6%、高于省定年度目标，完成84个黑臭水体治理。加快提升环境设施能力，全市危险废物集中焚烧年处置能力达10.3万吨，新增污水日处理能力5.5万吨。推进绿色转型，节能降耗完成省定目标，新增工信部绿色工厂7家、全省第二。提升生态绿城建设水平，建成老运河生态绿道和一批公园绿地，创成省生态园林城市。溧阳天目湖、金坛长荡湖湿地公园成为国家湿地公园，溧阳市荣获国家生态文明建设示范市称号。

　　（五）聚焦区域融合，城乡建设展现新面貌。主动融入长江经济带、长三角一体化发展等重大国家战略，突出区域协调、城乡统筹，城市集聚辐射带动力不断增强。高质量推进新一轮城市总体规划编制。加强交通基础设施建设，溧高、常宜、溧宁、苏锡常南部高速公路以及苏南沿江城际铁路工程加快推进，常泰过江通道开工建设，德胜河航道整治等重点项目进展顺利，233国道金坛段、360省道溧阳段等国省公路项目有序实施，京沪铁路常州站南北站房一体化改造工程竣工投用，新沟河延伸拓浚主体工程基本完成。加快完善城市路网，紫荆西路、河滨东路、大明路主线等项目竣工。地铁1号线实现“洞通”“轨通”“电通”，2号线土建工程过半。大运河文化带常州段规划建设加快推进，青果巷历史文化街区部分场馆完成修缮。公用基础设施加快建设，西石桥水厂、魏村水厂深度处理改造工程建成投运，完成天然气利用三期项目。城市长效管理深入推进，成为建筑垃圾资源化利用国家试点城市。实施交通畅行工程，新增停车位1.2万个，开辟校园定制公交和校车专线19条，投放有桩公共自行车2万辆。统筹推进特色田园乡村和全域美丽乡村建设，2个村庄获评中国美丽休闲乡村，7个村庄入选省特色田园乡村建设试点。加快完善农村基础设施，实施农村人居环境整治行动，农村污水设施覆盖率达80%。“四好农村路”省级示范市创建通过验收，溧阳创成“四好农村路”全国示范县，“1号公路”品牌进一步打响。创成全省首家全域省级节水型社会示范区。

　　（六）聚焦富民惠民，民生福祉获得新提升。始终坚持以人民为中心，积极回应群众关切，聚力解决影响百姓生活的“关键小事”。扎实推进就业创业，全年城镇新增就业13.23万人，扶持创业1.86万人，实现城乡就业困难人员再就业7700人。精准推进全民参保三年行动，社保扩面净增8.5万人。实施打赢打好脱贫攻坚战三年行动，茅山老区帮扶、“阳光扶贫”深入推进，在全省率先出台支出型贫困家庭生活救助办法，开展长期护理保险试点，慈善大病保险保障病种由4种扩大至30种，按照市定标准已脱贫建档立卡低收入人口1.84万户、3.26万人。大力改善百姓居住条件，11个易淹易涝片区整治工程全面完成，实施22万平方米危旧房安全隐患专项整治，棚改工程新开工24807套、基本建成18135套，积极推动既有住宅加装电梯。推进义务教育优质均衡发展，集团化办学覆盖率达66.86%；普高招生计划占比提高到56%；常州大学西太湖校区建成投用。综合医改持续深化，家庭医生签约服务质量不断提升，市妇幼保健院、市一院钟楼院区建成投用。扎实推进国家康复辅助器具产业综合创新试点，打造高品质现代健康养老“常州品牌”经验做法得到国务院领导批示肯定。文化惠民与精品创作、群众体育与竞技体育取得“双丰收”，建成243个基层综合性文化服务中心，推出滑稽戏《陈奂生的吃饭问题》等一批艺术精品，成功举办第十五届市运会，第十九届省运会金牌数列全省第三。持续推进全域文明城市建设，在全省首推文明行为信用管理，社会文明测评指数全省第一，溧阳市跻身全国新时代文明实践中心建设试点。加强和创新社会治理，在全省首创网格化服务管理中心与综治中心、合成维稳中心“三中心合一”模式，网格化社会治理走在全省前列；生产安全事故起数和死亡人数实现双下降；国家食品安全示范城市创建有力推进；深入开展扫黑除恶专项斗争，创成全域法治城市，社会大局保持和谐稳定。省双拥模范城创建再次实现全域“满堂红”。国防动员、人民防空、防震减灾、民族宗教、外事侨台、档案方志、妇女儿童、老龄、残疾人和关心下一代等工作取得新成效。

　　一年来，我们始终贯彻全面从严治党要求，切实加强政府自身建设。树牢“四个意识”，坚定“四个自信”，践行“两个维护”，坚决贯彻落实中央决策部署，开展习近平总书记对江苏重要指示批示贯彻落实情况“回头看”，认真落实中央巡视江苏反馈意见整改。扎实开展解放思想大讨论活动，市政府党组完成破解难题课题27个。深入推进作风建设，突出整治形式主义、官僚主义，推行首问负责制，政府效能得到提升。认真落实“一岗双责”，党风廉政建设和意识形态工作进一步加强。健全地方政府性债务监管制度体系，债务风险整体可控。加强审计监督和财政预算绩效管理，市级部门预算项目经费压减10%，市级机关闲置资产清理工作基本完成。坚持依法行政，市政府常务会议常态化学纪学法，提请市人大常委会审议地方性法规3件，颁布政府规章3件，行政综合执法体制改革深入实施。深化政务公开，举办政府开放日活动，加大政策解读宣传力度，基层政务公开标准化规范化试点工作通过国家级验收。坚持重大事项向市人大报告、与市政协民主协商，办理市人大代表议案建议201件、市政协提案343件。

　　各位代表，过去的一年，发展成就令人鼓舞。这是全市人民在中共常州市委正确领导下凝心聚力、团结奋斗的结果，是市人大、市政协加强监督、鼎力支持的结果。在此，我代表市人民政府，向辛勤工作在各个领域的全市人民，向市人大代表、政协委员，向市各民主党派、工商联、无党派人士、人民团体、各界人士和离退休老同志，向驻常人民解放军、武警官兵、公安政法干警和部省驻常单位，向所有关心、支持、参与常州建设和发展的海内外朋友们，致以崇高的敬意和衷心的感谢!

　　在肯定成绩的同时，我们也清醒地看到，我市推进高质量发展进程中还面临一些困难和问题。反映在外部环境上，经济下行压力有所加大，实体经济发展面临较大困难；中美贸易摩擦带来不确定性，直接影响外贸外资增长。反映在经济发展上，科技创新能力还不强，人才引育还不能适应转型发展需要，结构调整、产业升级任重道远；资源约束越发明显，环境保护任务艰巨。反映在社会民生上，公共服务资源配置还不够均衡，教育、医疗、养老等领域还有不少“痛点”“堵点”；社会治理面临一些新情况新问题，部分领域风险隐患不容忽视。反映在工作推进上，形式主义、官僚主义一定程度存在，抓落实的机制有待进一步完善。我们一定高度重视这些问题，采取有力有效措施，切实加以解决。

　　二、2019年工作安排

　　今年是新中国成立70周年，是决胜高水平全面建成小康社会的关键之年。今年政府工作的总体要求是：以习近平新时代中国特色社会主义思想为指导，深入贯彻党的十九大和十九届二中、三中全会精神，全面落实中央、省和市委的决策部署，坚持稳中求进工作总基调，坚持新发展理念，坚持推进高质量发展，坚持深化市场化改革、扩大高水平开放，以供给侧结构性改革为主线，以“重大项目攻坚年”活动为主题，统筹做好稳增长、促改革、调结构、惠民生、防风险各项工作，加快建设现代化经济体系，继续打好三大攻坚战，认真做好“六个稳”工作，全力种好幸福树、建好明星城，保持经济持续健康发展和社会大局稳定，以优异成绩庆祝中华人民共和国成立70周年。

　　做好今年工作，必须做到“六个更大力度”。一是更大力度稳定经济增长，保持稳中有进态势。牢记发展是第一要务、稳增长是第一目标，更大力度做好稳就业、稳金融、稳外贸、稳外资、稳投资、稳预期工作，充分发挥投资的关键作用、消费的基础作用、出口的支撑作用，努力克服各种不稳定不确定因素的影响，全力确保经济平稳运行。二是更大力度加快动能转换，提升经济发展质量。坚持把高质量发展作为根本要求，深入实施创新驱动和人才强市战略，强化新动能培育和传统动能改造提升，前瞻布局颠覆性技术、先导性产业，加快打造千亿级特色产业集群，不断提高资源节约集约利用水平和全要素生产率，实现更高质量、更有效率、更可持续的发展。三是更大力度深化改革开放，充分激发市场活力。坚持把改革开放作为根本路径，努力在政府自身改革、经济体制改革、城市建设管理体制改革等方面取得更大突破，全力打造国际化、法治化、便利化营商环境，持续激发市场活力和社会创造力。四是更大力度打好三大攻坚战，补齐发展突出短板。增强底线思维、落实守土责任，深入推进精准扶贫、精准脱贫，聚力打好污染防治攻坚战，抓好金融、政府隐性债务等领域风险防控，为决胜高水平全面建成小康社会提供有力支撑和保障。五是更大力度推进城乡融合，增强城市发展能级。坚持产城融合、区域协同、城乡一体发展，深度对接长江经济带、长三角一体化发展等国家战略，坚定不移走新型工业化、信息化、城镇化和农业现代化同步发展之路，扎实推进乡村振兴战略，全面提升城市综合竞争力。六是更大力度推进富民惠民，提高市民幸福指数。坚持以人民为中心，坚持尽力而为、量力而行，在保障底线民生的基础上，提升基本民生标准，追求高品质民生保障，不断满足人民日益增长的美好生活需要，让市民百姓因常州而自豪。

　　今年经济社会发展的主要预期目标是：

　　——经济支撑型指标夯实厚度。地区生产总值增长7%左右；一般公共预算收入增长6.5%左右；固定资产投资增长7%左右，制造业占比提高0.5个百分点左右；社会消费品零售总额增长8.5%左右；进出口总额增长3%左右；利用外资稳中提效，产业类占比达60%以上。

　　——质量结构型指标力求高度。全社会研发经费占地区生产总值比重达2.83%左右；科技进步贡献率达64%；高新技术产业产值占比达48%；新增高新技术企业300家；万人发明专利拥有量超35件；单位建设用地税收收入达5.19万元/亩，单位GDP建设用地占用率下降5.7%；制造业贷款新增80亿元。

　　——生态约束型指标瞄准精度。全市空气质量优良率达69%；PM2.5年均浓度降至49微克/立方米；城镇污水集中处理率达92%；林木覆盖率达26.5%；自然湿地保护率达50%以上；省考以上断面水质优Ⅲ类比例、节能降耗完成省定目标。

　　——文旅发展型指标拓宽广度。文化产业产值占GDP比重达5.8%；游客接待量增长10%左右，旅游总收入增长10%以上；推进36个新时代文明实践中心（所、站）建设试点，建好200个基层综合性文化服务中心。

　　——民生幸福型指标凸显温度。城镇新增就业与上年保持稳定、力争有所增长，城镇登记失业率控制在3%以内。全体居民人均可支配收入增长8%。居民消费价格涨幅控制在3%以下。

　　种好幸福树、建好明星城，是推动常州改革开放再出发、实现高质量发展的工作抓手和奋斗指向，是建设“强富美高”新常州的生动实践，是省委“六个高质量”发展要求的再聚焦、再落实、再提升。我们将聚力聚焦“种好幸福树、建好明星城”，围绕实现年度目标，重点抓好以下六个方面的工作：

　　（一）以加快动能转换为路径，推动经济发展高质量

　　围绕构建自主可控、特色鲜明的现代产业体系，深入推进供给侧结构性改革，用好人才第一资源，激发创新第一动力，加快培育以智能制造为标志的先进制造业、与先进制造业深度融合的现代服务业，全力塑造高质量工业明星城市。

　　强化项目带动。坚持把项目建设作为工作第一抓手、发展第一支撑，扎实开展“重大项目攻坚年”活动，以高质量投入带动高质量发展。突出产业链招商、产业集群招商，聚焦特色优势产业、传统产业高端环节、高端产业核心环节，坚持引资引技引智联动，精准对接央企和大院大所，在招引旗舰型、税源型、富民型重大项目和先导型、科技型精致项目上实现攻坚突破，力争全年招引30亿元或3亿美元以上重大突破项目17个。全力推进已签约重大项目落地开工，加快在建项目建设进度，抓好省、市重点产业项目建设，争取完成投资650亿元。积极破解土地要素制约，盘活存量建设用地2.5万亩、完成低效用地再开发1.2万亩。深化重大项目建设全周期管理，健全重大项目建设领导挂钩制，实施重大项目咨询论证和绩效评估，切实提升重大项目建设成效。

　　强化创新驱动。持续推进苏南国家自主创新示范区建设，加快建设长三角特色鲜明的产业技术创新中心。继续开展“科技长征”，深化与大学大院大所、领军人才团队合作，推动研发产业化。制定科教城新一轮发展规划，加快建设国际化产学研协同创新基地，加强与各产业园区的合作共建，更好发挥“创新之核”集聚和辐射带动作用。支持龙头企业牵头组建产业技术创新联盟，协同突破一批关键核心技术。加快引进建设重大创新平台和新型研发机构，重点建设新能源互联网、碳纤维和复合材料产业创新中心，以及中科院物理所长三角研究中心、中以产业技术研究院等。持续实施“创业常州”行动，推动建设一批特色众创空间。大力发展高新技术企业，打造更多创新型企业集群。深入实施“龙城英才计划”，以企业为主体大力引育海内外领军型创新创业人才，开展特色优势产业“人才特区”试点，带动引进各类高层次人才。推进创新创业友好型城市建设，对高薪酬类、高学历类、重点企业荐才类紧缺人才给予引才资助、购房补贴和租房补贴。探索建立领军人才企业自主创新产品政府首购首用制度。

　　强化智慧引领。精心举办好世界工业和能源互联网博览会，打造具有广泛影响力、辐射力的展会平台，推动智能制造企业加快集聚，促进智慧经济加快发展。实施“互联网+先进制造业”专项行动，支持创建一批省级以上工业和能源互联网平台。推进“千企上云”和机器换人，加快建设智能工厂，培育一批智能制造领军企业，全面提高企业智能化水平。实施“四新经济”培育工程，推动云计算、大数据、物联网、移动互联网、人工智能等加速发展，提升智能传感小镇、石墨烯小镇、瑞声科技小镇、无人机小镇等新经济集聚区发展质量。持续推进工业强基工程，提高先进制造业核心竞争力。深入实施常州质量提升行动，推动创建全国知名品牌示范区、优质产品生产示范区和质量认证示范区（点）。加强知识产权保护，支持企业更多参与制定国际标准、国家标准和行业标准，增强市场控制力和行业话语权。

　　强化产业集聚。制定实施特色产业集群建设行动计划，重点在智能装备制造、以石墨烯和碳纤维为代表的新材料、新一代信息技术等领域打造全国领先的产业标杆，形成规模优势明显、核心技术领先、产业配套完善的产业集群。进一步优化产业空间布局，推动辖市区分别打造2-3个特色产业集群。鼓励支持龙头企业发展壮大，扶持培育专精特新“小巨人”企业，加快形成大中小企业分工协作的产业生态体系，促进全产业链整体跃升。加快建设军民融合公共服务平台，积极培育军民融合特色产业集群，争创国家级军民融合示范基地。

　　强化产业协同。着眼支撑制造业高端攀升和满足群众高品质生活需求，着力发展科技服务、软件和信息、工业设计、人力资源、现代物流等生产性服务业，推动生产型制造企业向服务型制造企业转型。把更好服务实体经济与防范化解金融风险有效结合起来，推进金融产品创新，建好企业征信、综合金融等服务平台，积极发展产业投资引导基金、股权投资基金，引导信贷资金更多流向先进制造业，推动解决中小企业融资难融资贵问题，稳妥处理上市公司股票质押风险、中小企业融资互保链风险。大力推进企业股改上市，促进科技型企业拥抱科创板，全年新增上市公司10家、股份制企业100家。把消费对经济的拉动放在更加突出的位置，大力促进消费升级，加快教育医疗、育幼养老、文化旅游等服务业发展，挖掘新兴消费潜力，切实改善消费环境，落实好个人所得税专项附加扣除政策，让百姓吃得放心、穿得称心、用得舒心。

　　（二）以推进文旅融合为抓手，推动文化建设高质量

　　围绕文化名城建设、国家全域旅游示范区创建和休闲旅游目的地建设，双向融合“旅游+”与“+旅游”，进一步传承历史文化、彰显山水特色、打响旅游品牌，全力塑造高质量旅游明星城市。

　　深度融合文化与旅游。加快实施大运河文化带常州段总体规划，着力推进怀德桥至东坡公园精华段建设，重点实施南市河历史文化街区“微改造”，提升前后北岸历史街区文化内涵，建成开放青果巷历史文化街区。推进孟河、焦溪、杨桥等古镇古村保护，实施意园、夏家大院修缮保护工程。有序实施历史城区更新改造，打造有历史韵味的“城市客厅”，建设一批“建筑可阅读”的微旅行街区。加强红色文化资源的保护利用，塑造以“常州三杰”为核心的红色文化旅游品牌。提炼“龙文化”丰富内涵，提升“龙城象教”等文旅品牌价值。

　　提升休闲度假旅游品质。推动天目湖、太湖湾、茅山、长荡湖、曹山等旅游度假区提档升级，启动西太湖旅游度假区开发建设；着力培育南山竹海、茅山闲养、嘉泽园艺等一批旅游风情小镇和查家湾、仙姑村等一批乡村旅游特色村，打响“度假胜地”品牌。推进环球恐龙城、淹城春秋乐园、动漫嬉戏谷等主题公园创新提升和错位发展，实施东方盐湖城二期项目，增强主题公园核心竞争力，打响“乐园之都”品牌。制定实施主城区餐饮业提升三年行动计划，重塑南大街、双桂坊等经典旅游餐饮街区，持续开展常州美食评比推介活动，让“食美常州”成为旅游明星城市的独特标签。促进各类资源与旅游业对接，加快打造体育旅游、工业旅游、研学旅游、康养旅游、非遗旅游等新业态，支持旅游大数据平台建设，推动智慧旅游发展。

　　促进文化事业大发展。鼓励和支持文艺精品创作，大力发展新兴文化业态，推动骨干企业做大做强，支持小微文化企业发展壮大，提升文化创意产业集聚区发展水平。积极创建国家公共文化服务体系示范区和省“书香城市”示范市，建成常州文化广场，实现基层综合性文化服务中心全覆盖，举办好“社区天天乐”“文化100”“文化点亮生活”等惠民活动。

　　（三）以增强发展活力为目标，推动改革开放高质量

　　坚持向改革要动力、向开放要活力，聚焦重点领域全面深化改革，围绕高质量发展扩大对外开放，进一步优化经济社会发展环境，全力塑造高质量管理明星城市。

　　持续推动政府职能转变。坚持刀刃向内，稳步推进政府机构改革，全面优化职能配置，不断创优政务管理。继续深化“放管服”改革，加快打造“四通四办四平台”审批服务新模式，推进企业投资项目承诺制试点，支持新北区推进集成改革。以“一网通办”为抓手，更大力度推进政务信息整合共享和大数据融合利用，高质量实现“不见面审批”，让企业和群众办事像网购一样便捷。坚持政府少干预、市场多配置，凡是市场能自主调节的就让市场来调节，凡是企业能干的就让企业干。

　　充分激发企业发展活力。毫不动摇支持民营经济发展，加快破除制约微观主体活力释放的体制机制障碍，全面落实进一步降低企业负担促进实体经济高质量发展的各项政策措施，确保民营企业依法平等使用生产要素、公平参与市场竞争、同等受到法律保护。加强企业家队伍建设，激发和保护好企业家精神、工匠精神。深入推进国资国企改革，推动国有企业实施差异化发展战略，加快做强做优。进一步优化国有资本布局，推动国有企业全面建立现代企业制度，以股改上市为主要形式稳步实施混合所有制改革，加快将机关事业单位所属企业的国有资本全部纳入统一监管体系。

　　切实创新社会治理。深入实施六大文明创建行动，注重运用先进文化锤炼城市精神气质，推进新时代文明实践中心（所、站）建设试点，提升道德讲堂等特色文明品牌。打造全要素、全市域、智能化、大联动网格化社会治理模式，确保网格化创建达标率居全省前列。深化平安常州建设，完善现代治安防控体系，健全合成维稳、消防安全管理、应急管理新机制，深入推进扫黑除恶专项斗争。切实抓好安全生产，加强食品药品安全监管。提升国防动员能力和双拥优抚层次，巩固发展军政军民团结。加强法治常州建设，深化“互联网+”普法活动。高度重视信访工作，妥善化解社会矛盾，确保社会大局和谐稳定。

　　加快提升开放能级。主动融入全省高质量建设“一带一路”交汇点大格局，增创对外开放新优势。以“一特三提升”为导向推动各类园区转型创新，推动常州、金坛经开区和江苏中关村升格国家级。深入推进工业企业资源集约利用综合评价，制定差别化政策，倒逼低效、落后产能退出。大力推进中以常州创新园合作共建，打造以色列技术、项目、资本在国内合作的“桥头堡”和示范区。高起点规划建设苏澳合作园区，争取区域金融改革试点，打造“一国两制”框架下澳门与内地融合创新试验区。加强与中科院对接合作，争取国家空间信息综合应用创新服务平台落户。提升中德、中瑞等中外合作园区建设水平。积极复制自贸区试点经验，促进投资贸易便利化，推动综合保税区开放升级，提高先进制造业利用外资比重。持续优化外贸结构，加快培育重点出口企业、出口产品，支持外贸企业开拓新兴市场。支持企业参与“一带一路”建设，鼓励本土企业跨国并购，布局全球产业链。

　　（四）以建设精美常州为导向，推动城乡建设高质量

　　坚持以“四化同步”为指引，以产城融合发展、城乡融合发展为方向，加快推进以人为核心的新型城镇化，深入实施乡村振兴战略，全力打造具有突出影响力的长三角区域中心城市。

　　加快重大基础设施建设。围绕打造“江苏中轴”枢纽城市，加快构建开放型综合立体交通网络，重点推进常宜、苏锡常南部通道、溧高、溧宁高速公路以及常泰过江通道、苏南沿江铁路、德胜河三级航道整治等重点工程建设。加快新孟河延伸拓浚等水利工程建设，新沟河工程全面完工。完善轨道交通线网规划，地铁2号线加快建设，1号线建成投运，常州阔步迈入“地铁时代”。加快常州综合港务区建设步伐，推动常州国际机场加快实施航站楼改扩建工程。进一步完善城市路网，建成新机场路快速化改造一期、星港西路西延等项目。

　　扎实推进城市精细化管理。以绣花精神治理城市，深化城市长效管理和城乡一体化考评，推进数字城管向智慧城管升级。深入实施交通畅行工程，推广智慧停车和定制“学生专车”，加强电动车、机动车违停综合治理，全面优化公交线网，积极创建国家公交都市。加强市容环境卫生管理，开展地铁1号线沿线市容环境综合整治，推动创建省城市管理示范路和示范社区。建立违法建设治理长效机制，推进住宅物业管理提升和垃圾分类治理，继续改造易淹易涝片区，打造一批宜居示范小区。

　　深入实施乡村振兴战略。全面提升农业现代化水平，加快实施“310”发展计划，着力推动十大现代农业产业示范园建设、农业企业股改上市、农产品知名品牌培育。不断增加优质绿色农产品供给，强化粮食安全责任，实现千家农产品生产主体质量追溯，建设百家现代农业标准化示范基地，提升十大农产品市场。持续推进农村集体产权制度改革，力争90%以上的村完成改革任务。积极培育家庭农场、农民合作社等新型经营主体，实施百名农业精英人才培育工程。启动全域美丽乡村建设，建好美丽乡村示范区（点），打造万亩美丽休闲农田，培育农村土地资源统筹配置示范点，全力开展好“大棚房”问题专项清理整治行动。深入实施农村人居环境整治，提升农村宜居环境。继续推进“四好农村路”建设，构建全域旅游农村公路网。

　　持续推动区域协同发展。把提升县区经济摆到重要位置。鼓励县区突出特色、错位发展，支持溧阳开展社会主义现代化建设试点、金坛建设城乡融合发展试验区、武进建设“苏南模式”转型升级试验区、中心城区落实精明增长理念，加快提升综合竞争力。促进县区联动发展，继续推动产业、科技、项目、基础设施“四个西进”，推进天宁开发区“东扩”和钟楼开发区“西进”，提升全市域协同发展水平。加强与周边城市的协同联动，主动融入上海大都市圈，深化苏锡常一体化、锡常泰跨江融合发展，鼓励支持溧阳、金坛融入南京都市圈，溧阳建设苏皖合作示范区。

　　（五）以攻坚污染防治为重点，推动生态环境高质量

　　牢固树立“绿水青山就是金山银山”理念，坚决打好污染防治攻坚战，加快解决突出环境问题，促进经济发展与生态保护共赢。

　　全力攻坚污染防治。扎实推进中央、省环保督察反馈问题整改，实施大气污染防治攻坚行动，强化重点污染源、“散乱污”企业、扬尘污染、机动车和船舶污染、挥发性有机物治理，狠抓长江经济带化工污染专项整治，扩大高污染车辆禁限行区域、路段，加强市区烟花爆竹禁放管理，确保PM2.5和臭氧浓度“双控双降”。严格落实湖长、河长、断面长责任，继续实施畅流活水工程，建设“五好”示范河道，确保国省考核断面水质达到考核要求，基本消除全市域黑臭水体。实施土壤污染防治行动计划，推进土壤治理修复及风险管控。以更实举措调整产业结构、能源结构和运输结构，加快化工钢铁煤电印染行业转型升级，完成东部热电整合。

　　全面加强生态建设。实施郊野公园、城镇公园绿地、生态绿道等建设工程，建设大运河文化带生态长廊，新增一批群众身边的小游园、健身设施，积极创建国家生态园林城市。加强长江岸线资源保护与利用，持续保护和修复长江重要湿地等生态系统，加快建设沿江绿色生态廊道、滨江风光带，积极创建长江大保护绿色转型发展先行实验区。强化太湖水域生态修复，推动武进生态保护引领区建设，实现更高水平“两个确保”。

　　健全生态保障体系。完善监管执法体系，加大明查暗访、媒体曝光力度，以零容忍态度依法严惩环境违法行为。落实“三线一单”，实行生态环境损害赔偿，完善生态环境财政奖补机制。全面实施超低排放企业限停产豁免制度，支持和鼓励企业瞄准“超低排放”目标，加大技改力度，打造绿色标杆企业。加快构建天地空一体的生态环境监测网络，提升污染防治科学化、精准化水平。推进生活垃圾、建筑垃圾、工业固废和危险废物处置设施重点项目建设，加快补齐污染防治设施短板。

　　（六）以增进民生福祉为根本，推动人民生活高质量

　　坚持以人民为中心的发展思想，认真落实“坚守底线、突出重点、完善制度、引导预期”的要求，聚焦群众“关键小事”，着力办好民生实事，不断增强群众的获得感幸福感安全感。

　　持续促进富民增收。坚持就业优先战略，实施大学生就业创业促进计划、“技能龙城”建设计划，保障好大学生、农民工、退役军人等重点群体就业，开展职业技能补贴培训2.6万人次。完善职工工资正常增长机制，拓宽居民财产性收入渠道。坚持阳光扶贫、精准脱贫，深入实施茅山老区“百千万”帮扶工程，推动经济薄弱村实现年收入100万元目标，加大特殊贫困群体脱贫攻坚力度，基本完成全市建档立卡低收入人口脱贫任务。做好对口扶贫协作工作，推动优势互补、共同发展。

　　坚持教育优先发展。进一步优化教育资源布局，实施北郊初中改扩建等50个学校建设项目，清潭中学南校区等27个项目竣工投用，增加学位1.87万个。持续推进义务教育集团化办学，扩大优质教育覆盖面。普遍开展中小学生课后服务，关心学生身心健康。大力支持公办、民办普惠性幼儿园发展，多渠道解决“入园难”问题。加快普通高中师资队伍建设，继续增加普通高中招生计划。支持在常高校提高办学和科研水平，推进产教融合发展，南航天目湖校区竣工投用。

　　聚力建设健康常州。深化“健康常州”建设，扩大综合医改成果。加强基层医疗卫生体系和全科医生队伍建设，启动县域医共体建设，全面提升家庭医生签约服务质量。实施市七院二期、市二院阳湖院区二期、市四院肿瘤病房大楼等工程，大力发展“互联网+医疗健康”，推进中医药传承创新，增加优质医疗资源供给。以“三项融合”为特色，打响健康养老的常州品牌。引导民间资本进入养老领域，社会力量举办或经营的养老床位占比达70%以上。加强医养联合体建设，实施百家养老院质量提升行动，加快住宅小区适老化改造，支持老旧小区加装电梯。大力发展体育事业，积极推动体医、体教融合，全力争创国家全民运动健身模范市。

　　完善提升社会保障。推进社会救助政策与扶贫开发政策有效衔接，提升救助精准度。研究制定重特大医疗疾病救助补充办法，慈善大病保险扩面到一般家庭。完善住房市场和住房保障体系，推进公共租赁住房货币化保障，住房公积金扩面10万人，新开工棚户区改造1.6万套（户）。稳步提高企业退休人员基本养老金、城乡居民基础养老金待遇标准。深入推进全民参保登记工作，推动社会保障从制度全覆盖向人群全覆盖转变。加强妇女和未成年人权益保护，发展残疾人事业。

　　根据市委关于推行市民生实事项目人大代表票决制工作的实施意见，市政府从市民群众、人大代表、政协委员和辖市区、市级部门等层面广泛征集，遴选形成12个候选项目。经本次人代会票决，10个项目确定为市政府2019年度民生实事项目。对民生实事项目，市政府将加强组织领导、严格责任考核、强化统筹协同、狠抓工作落实，并接受市人大代表和社会各界监督，确保实事办好、好事办实。

　　三、全面建设人民满意政府

　　常州正处在发展的转型期、关键期。越是在转型升级压力大的时候越要保持定力、坚定信心，越是在困难矛盾特别多的时候越要勇于担当、主动作为，这是我们的职责所在。我们将以习近平新时代中国特色社会主义思想为指引，认真贯彻全面从严治党要求，切实增强政府系统的执行力、公信力、战斗力。

　　一是加强思想政治引领。更高标准加强政治建设，旗帜鲜明讲政治，牢固树立“四个意识”，坚定“四个自信”，忠诚践行“两个维护”，始终与以习近平同志为核心的党中央保持高度一致。自觉把党对一切工作的领导贯彻到政府工作各领域各方面，确保中央大政方针和省委省政府、市委决策部署不折不扣落地见效，抓紧抓实中央巡视江苏、省委巡视常州反馈意见整改。更深层次推进思想解放，认真落实意识形态工作责任制，为高质量发展汇聚强大正能量。

　　二是推进全面依法行政。全面贯彻依法治国基本方略，坚持法定职责必须为、法无授权不可为，尊法学法守法用法，加强法治政府建设。完善政府立法机制，深化综合行政执法体制改革，全面落实“双随机一公开”监管。自觉接受市人大及其常委会法律监督、工作监督和市政协民主监督，以及司法监督、群众监督、舆论监督，广泛听取各民主党派、工商联、无党派人士和人民团体的意见，认真办理人大代表议案建议和政协提案。深化政务公开，让权力在阳光下运行，让透明成为常态。

　　三是激励干部担当作为。进一步推行政府工作项目化，完善抓落实机制，确保各项任务落到实处。推动各级政府诚信施政，带动全社会讲诚信守信用。按照“坚定、可控、有序、适度”的要求，稳妥化解政府性债务风险，坚决杜绝违规变相举债行为。推进作风建设常态化制度化，落实鼓励激励、容错纠错、能上能下“三项机制”，加大对工作成效明显的地区（部门）督查激励力度，坚决整治形式主义、官僚主义，用好问责利器，大力营造干事创业、勇争一流的良好氛围。

　　四是提升政府服务水平。推动政府系统党员干部争做学习型干部、开展研究型工作，增强工作的主动性、系统性和专业性，提高谋划和推动工作的能力，应对复杂局面、解决实际问题的能力，服务企业和服务群众的能力。严格落实首问负责制，以“我的常州”APP等信息化手段，为群众和企业提供优质高效服务。大力弘扬“店小二”精神，提升常州政企通“一网一群一号”服务功能，着力构建亲清新型政商关系，让每一位企业家都能在常州放心投资、安心创业、舒心生活。

　　五是深化反腐倡廉建设。坚决落实全面从严治党各项要求，健全廉政风险防控机制。加强国有资产资源、民生环保等领域审计监督，强化政策执行审计。管好用好公共财政资金，市级预算单位公用支出定额再压降10%；深化市级政府专项资金管理，市级财政涉企专项资金项目实行统一平台申报。加强市级党政机关及其下属单位用房集中统一管理。深入推进政府系统反腐败斗争，严格落实中央八项规定及实施细则，严肃查处群众身边“四风”和腐败问题，树立清正廉洁良好形象。

　　各位代表，常州发展的历史使命，激励我们砥砺奋进；全市人民的信任期盼，鞭策我们勇往直前。让我们更加紧密地团结在以习近平同志为核心的党中央周围，在省委省政府和市委的坚强领导下，解放思想、锐意进取，攻坚克难、真抓实干，以奋斗姿态种好幸福树、建好明星城，推动常州高质量发展走在前列，以优异成绩向新中国成立70周年献礼！
